# Endothelial cells release microvesicles that harbour multivesicular bodies and secrete exosomes

**DOI:** 10.1002/jex2.79

**Published:** 2023-03-30

**Authors:** Jennifer D. Petersen, Elena Mekhedov, Sukhbir Kaur, David D. Roberts, Joshua Zimmerberg

**Affiliations:** ^1^ Section on Integrative Biophysics, Division of Basic and Translational Biophysics, Eunice Kennedy Shriver National Institute of Child Health and Human Development National Institutes of Health Bethesda Maryland USA; ^2^ Laboratory of Pathology, Center for Cancer Research, National Cancer Institute National Institutes of Health Bethesda Maryland USA

**Keywords:** electron microscopy, endothelial cell, exosome, Extracellular vesicle, intraluminal vesicle, microvesicle, multivesicular body, protrusion

## Abstract

Extracellular vesicles (EVs) released by endothelial cells support vascular homeostasis. To better understand endothelial cell EV biogenesis, we examined cultured human umbilical vein endothelial cells (HUVECs) prepared by rapid freezing, freeze‐substitution, and serial thin section electron microscopy (EM). Thin sections of HUVECs revealed clusters of membrane protrusions on the otherwise smooth cell surface. The protrusions contained membrane‐bound organelles, including multivesicular bodies (MVBs), and appeared to be on the verge of pinching off to form microvesicles. Beyond cell peripheries, membrane‐bound vesicles with internal MVBs were observed, and serial sections confirmed that they were not connected to cells. These observations are consistent with the notion that these multi‐compartmented microvesicles (MCMVs) pinch‐off from protrusions. Remarkably, omega figures formed by fusion of vesicles with the MCMV limiting membrane were directly observed, apparently releasing exosomes from the MCMV. In summary, MCMVs are a novel form of EV that bud from membrane protrusions on the HUVEC surface, contain MVBs and release exosomes. These observations suggest that exosomes can be harboured within and released from transiting microvesicles after departure from the parent cell, constituting a new site of exosome biogenesis occurring from endothelial and potentially additional cell types.

## 1 INTRODUCTION

Signalling between cells mediated by secreted membrane‐enclosed organelles called extracellular vesicles (EVs) is a widespread form of intercellular communication, evolutionarily conserved from bacteria to plants and animals (Colombo et al., 2014; Gill et al., 2019). Cells load EVs with a range of bioactive cargos including lipids, membrane proteins, adhesion proteins, cytoskeletal elements, enzymes, signalling molecules, and nucleic acids. Once released into the extracellular milieu, EVs can signal locally or travel long distances in body fluids—such as blood, lymph, cerebrospinal fluid, amniotic fluid—to act on remote tissue targets. Upon reaching recipient cells, specific interactions between EVs and target cells promote binding and uptake by pinocytosis, phagocytosis, endocytosis, or direct fusion with the plasma membrane (Gurung et al., 2021; van Niel et al., 2018). EV‐mediated intercellular signalling is a ubiquitous mechanism occurring under physiological and disease states (Cheng & Hill, 2022; Isola & Chen, 2017). Plasma EVs are proving to be sensitive biomarkers of numerous disease states, such as cancer, that can be obtained by a liquid biopsy (Mitchell et al., 2022). Furthermore, EVs are being developed as vehicles for delivery of therapeutic agents (Sil et al., 2020). Despite these important physiological functions and medical utilities, much remains to be discovered about the biosynthesis of EVs.

It is generally viewed that EVs fall into two categories based on their site of biogenesis. Microvesicles arise at the plasma membrane, by outward budding and pinching off directly from the cell surface. In contrast, exosomes are of endosomal origin, produced when intraluminal vesicles (ILVs) contained in multivesicular bodies (MVBs) are exocytosed upon MVB fusion with the plasma membrane (Mathieu et al., 2019; van Niel, D'Angelo & Raposo, 2018). Microvesicles range in diameter from 50 nm to over 1 μm and include ectosomes, microparticles, shedding vesicles, migrasomes, and oncosomes (if released from cancer cells). Apoptotic bodies are an additional type of microvesicle, formed when a cell undergoing programmed cell death breaks up into 500 nm – 5 micron‐diameter fragments with limiting membranes derived from the cell's plasma membrane (Battistelli & Falcieri, 2020; Xu et al., 2019). In accord with the idea that exosomes represent ILVs that have been released by exocytosis from MVBs, they exhibit a narrower size range between 40 and 150 nm (Kang et al., 2021).

Endothelial cells form the endothelium, a single cell‐thick lining of the blood and lymphatic vessels that controls the exchange of oxygen and nutrients between the vessel contents and underlying tissues (Ricard et al., 2021). In their role on the “front lines” of vessels, exposed to circulating cells and plasma, endothelial cells release a significant proportion of the EVs found in blood (Mathiesen et al., 2021). EVs released by the endothelium contribute to its role in supporting vascular homeostasis, which includes maintenance of the antithrombogenic surface of the vessels (blood fluidity) and vasodilation, inhibition of inflammation, cell survival and angiogenesis (Trisko et al., 2022).

To gain a better understanding of the mechanisms and structural aspects of EV release from endothelial cells under pro‐angiogenic but noninflammatory conditions, we used thin section electron microscopy (EM) to examine Human Umbilical Vein Endothelial Cells (HUVECs) and look for structural features consistent with microvesicle budding from the plasma membrane and exocytic release of exosomes from MVBs. Cells were preserved by ultra‐fast freezing, which is optimal for capturing fast events like exocytosis, and processed by a freeze substitution protocol optimized for plasma membrane enhancement (Walther & Ziegler, 2002).

In thin sections, groups of protrusions were observed on the otherwise smooth HUVEC plasma membrane that were often branched and contained vesicular organelles, including MVBs with ILVs. Beyond cell peripheries, vesicles that contained MVB‐like vesicles were observed, suggesting that they were microvesicles that had pinched off from the protrusions, diffused, and occasionally adhered to the coverslip. Serial sections through the presumptive microvesicles on the coverslip confirmed that they were not connected to cells by cellular extensions and that ILV‐like vesicles were within the MVB‐like vesicles. Further examination revealed omega figures, the structural hallmarks of exocytosis (Douglas, 1973), occurring between MVB‐like vesicles inside the microvesicles and their limiting membrane. On occasion, such omega figures contained small vesicles that were identical to ILVs. These observations support the notion that microvesicles containing multiple membrane compartments (referred to as multi‐compartmented microvesicles, or MCMVs) pinch off from MVB‐containing protrusions at specialized sites on the cell surface. MCMVs contain MVBs that apparently can release exosomes after transiting away from the parent cell.

## 2 METHODS AND MATERIALS

### 2.1 HUVEC culture on Aclar coverslips for thin section EM

HUVECs were obtained from American Type Culture Collection (ATCC, Manassas, VA, USA; #PSC‐100‐013). HUVECs between passages 4–6 were used in this study, screened for mycoplasma contamination (ATCC, Kit #30‐1012K), and cytotoxicity (ThermoFisher, Cat. No. L3224) and analyzed at a subconfluent density. Cells were plated on Aclar 33C plastic film coverslips (Electron Microscopy Sciences [EMS], Hatfield, PA, USA). Compared to glass coverslips which adhere strongly to embedding resin used for thin section EM, Aclar 33C plastic separates easily and cleanly from embedding resin without scratching or damaging the surface of the resin, which corresponds to the surface of the coverslip where the cells are adhered (Kingsley & Cole, 1988). To plate HUVECs on Aclar, the plastic sheet of Aclar was cut into about 12 mm‐wide rectangular coverslips to fit into wells of a 12‐well plate using clean scissors. After cutting into desired size and shape, coverslips were washed at least ten times with distilled water and then at least ten times with 70% ethanol. Coverslips were then placed into wells of a 12‐well plate and rinsed at least ten times with cell culture grade sterile water (ThermoFisher Scientific, Waltham, MA, USA), and then once with endothelial cell culture medium (ScienCell, Carlsbad, CA, USA, Cat. No. 1001) which contains 5% fetal bovine serum, 1% endothelial cell growth supplement, and 100 U/ml Penicillin/Streptomycin antibiotic solution. Cells were plated (30,000 cells per well) and maintained for 2–3 days in an incubator at 37°C and 5% CO_2_. Medium was changed every 48 h.

### 2.2 Plunge freezing of cultured HUVECs

To preserve cells for EM, cultured HUVECs on Aclar coverslips were plunge frozen by hand in liquid ethane. Ethane was liquified in the “ethane pot” of a Vitrobot Mark IV plunge freezer (ThermoFisher Scientific) following manufacturer's instructions. A vessel of liquid nitrogen was placed next to the ethane pot for transfer of frozen coverslips to storage containers under liquid nitrogen. To freeze, a coverslip was gently withdrawn from well of 12‐well plate with pair of clean, fine‐tipped forceps (Dumont #5, EMS). Holding the coverslip vertically, excess culture medium was wicked away from the edges of the coverslip using Whatman #1 filter paper (EMS). Total blotting time did not exceed 5 s, with careful observation to avoid drying. After blotting, the coverslip was inverted over the liquid ethane, and plunged into the liquid ethane by hand as quickly as possible. The coverslip was immediately transferred to liquid nitrogen and stored under liquid nitrogen until processing by freeze substitution.

### 2.3 Freeze‐substitution of cultured HUVECs on Aclar coverslips

Frozen cells were fixed by freeze substitution, a process that dissolves and replaces ice in the tissue by a solvent while at low temperature, and in the presence of fixatives (Feder & Sidman, 1958). The freeze substitution (FS) process used was a slightly modified version of a protocol developed to enhance preservation and staining of lipid membranes (Walther & Ziegler, 2002). FS staining cocktail was prepared in 20 ml glass scintillation vials with plug caps (EMS) and contained: 0.2% osmium tetroxide, 0.1% uranyl acetate (UA) in glass distilled acetone with 5% water. To prepare the FS cocktail, working under a fume hood, 12 mg of UA (EMS) was added per vial, followed by 600 μl of 4% aqueous osmium (EMS) and vortexed for 30 s to dissolve. Then, 11.4 ml of acetone from a freshly opened bottle of glass distilled acetone (EMS) was added to each vial. The vial was tightly capped, and contents mixed well by swirling. Then caps were loosened and placed in a liquid nitrogen bath until the FS cocktail was frozen solid. Liquid nitrogen was poured directly in the vials, and then frozen coverslips bearing cells were transferred into vials while being maintained under liquid nitrogen. One coverslip was processed per vial. Vials were loosely capped and transferred to the chamber of the automatic freeze substitution machine (EM AFSII, Leica Microsystems, Wetzlar, Germany) that contained an approximately 15 mm‐deep bath of ethanol to improve thermal conductivity, and was pre‐cooled to −112°C. Within about 10 min in the freeze substitution machine, the liquid nitrogen inside the vials was evaporated and vial caps were tightened. The following FS program was applied: warm from −112°C to −90°C, then hold at −90°C for 8 h, then warm to 20°C for 36 h.

### 2.4 Room temperature processing and embedding of freeze‐substituted HUVECs

When the FS program was completed, vials were removed from the machine and processed at room temperature. Coverslips were rinsed with acetone three times and then stained with 0.5% tannic acid in acetone for 45 min, protected from light. Tannic acid was removed by four acetone rinses using freshly opened bottle of glass distilled acetone. Cells were infiltrated with a graded series of EmBED812 resin (EMS) in acetone (50%, 75%, 95%, and 100% resin twice.) Each infiltration step lasted for at least 1 h. The coverslips were embedded as follows: a square “backing piece” of Aclar was cut into a square and laid flat into either the lid or base of a 35 mm plastic Petri dish, which serves as a carrier for the embedding assembly. Then, the coverslip to be embedded was removed from the scintillation vial with clean forceps, and excess resin was allowed to drain from the coverslip for a few seconds. Then the coverslip was placed cell‐side‐up on the Aclar backing piece in the Petri dish. A gelatin capsule, with the cap removed, (EMS, size 0) was filled with freshly prepared 100% resin until it was level with the rim of the open capsule, and any bubbles were allowed to float to the surface and removed. Then the uncapped, resin‐containing gelatin capsule was inverted and placed directly on top of the cell‐side of the coverslip. Two or three gelatin capsules were placed side‐by‐side to cover most of the surface of the coverslip, and the entire assembly was put into the 60°C oven for 48 h to polymerize.

### 2.5 Serial thin sectioning of embedded HUVECs

After polymerization, the backing piece of Aclar was removed, and the gelatin capsules attached to coverslips were separated from each other with a jewelers saw into individual blocks. Often the gelatin capsule could be sawed into three to four pieces to allow sectioning of multiple areas per block. After trimming away excess resin with a double‐edged razor blade, the Aclar was removed by inserting the edge of the razor blade at a corner, between the Aclar and the resin surface, and lifting carefully to flick away the Aclar coverslip, revealing the smooth bottom surface of embedded cells. The block face was then trimmed to an approximately 600 μm wide × 400 μm tall trapezoid shape for sectioning. Sections were cut using an ultramicrotome (EM UC7, Leica Microsystems) to a thickness of 70 nm using a diamond knife (DiATOME, Hatfield, PA, USA). Typically, four to five serial sections were positioned with an eyelash brush and picked up per 2 × 1 mm slot grid that was formvar and carbon coated (EMS). Because the Aclar coverslip, and therefore the resin surface, was not perfectly flat, and inevitably the knife edge was not perfectly aligned with the block face, the knife grazed portions of the block face (which corresponded to the coverslip surface) and missed other portions of the block face at first, with successive sections becoming more complete until the knife fully entered the resin. The partial sections were collected and used to determine the distance from the coverslip of structures imaged in serially collected thin sections. For instance, if a structure was present near the grazed edge of a partial section where the knife first entered the resin, and that area of resin was not yet cut in the previous section, then it could be ascertained as the first 70 nm‐thick section in which the structure appeared, corresponding to the surface of the coverslip. Adjacent sections could then be examined, in order, each containing the next 70 nm slice of the structure. Sections on the grids were post‐stained with 3% Reynolds lead citrate (EMS) for 5 min.

### 2.6 Chemical fixation and EM processing of HUVECs

All steps were performed at room temperature, and all reagents were obtained from EMS unless otherwise stated. HUVECs grown on glass or Aclar coverslips were fixed with 2% paraformaldehyde and 2% glutaraldehyde in 0.1 M sodium cacodylate buffer containing 2 mM calcium chloride for at least 60 min. After fixation, cells were rinsed with cacodylate buffer and then postfixed for 60 min with 0.25% osmium and 0.25% potassium ferrocyanide (Fisher Scientific, Pittsburgh, PA, USA) in cacodylate buffer. After rinsing with cacodylate buffer, cells were incubated in 0.5% tannic acid in cacodylate buffer for 30 min, rinsed with acetate buffer at pH 5.2, and then stained with 2% uranyl acetate in acetate buffer for 60 min. Finally, cells were dehydrated, embedded, and sectioned as described above for the freeze substituted HUVECs.

### 2.7 Transmission EM of serial thin sections and image processing

Thin sections were viewed using a Tecnai T20 transmission electron microscope (ThermoFisher Scientific) operated at 200 KeV. When a structure of interest was discovered that spanned multiple sections, the structure was tracked back in the serial sections to the first section in which it appeared, and then imaged in consecutive sections moving up in the z‐axis away from the coverslip. Images were collected with an NanoSprint1200 side‐mount CMOS detector (Advanced Microscopy Techniques, Woburn, MA, USA). Adobe Photoshop 2022 (San Jose, CA, USA) was used to adjust images for display by applying a Gaussian blur of 0.5 pixel‐radius, followed by adjustment of grey levels, brightness, and contrast. Serial section images were aligned using the TrakEM2 plugin of Fiji image analysis software (Schindelin et al., 2012) which is a version of ImageJ2 (Rueden et al., 2017) equipped with additional plugins for image analysis. Fiji is available as an open‐source software (https://imagej.net/software/fiji/downloads).

### 2.8 Quantification of structures in EM thin sections

To quantify the proportion of cells with protrusion sites from individual thin sections, cut from at least two independent blocks of resin prepared from coverslips of three independent cultures were examined. The number of total cells found in these thin sections, and the fraction of cells with and without protrusion sites were counted (omitting partial cells on the edge of sections). Because we could not see the entire membrane, we constructed a simplified model to adjust to the total cell membrane. We estimated the total cell membrane area to be about 1200 μm^2^ using a pancake‐like model based on a vertical section showing the height of the HUVEC (∼4 μm) and a mean diameter of 24 μm (Chen et al., 2017). Because in the thin section (X‐Y plane) the protrusion site base measured 2–8 μm wide (see Supplemental Figure S1) we chose 2 μm as a conservative measure of the extent of a protrusion site in Z. Then, 2 μm worth of serial sections would show the same protrusion site. But only 9% of the thin section cells showed even one protrusion site. The ratio of the 2 μm band of surface (which for a 24 μm cell has ∼150 μm^2^) to the total cell surface (1200 μm^2^) gives the mean number of about ¾ protrusion sites per cell.

Similarly, the frequency of MVB‐like vesicles inside MCMVs and omega figures observed on the periphery of MCMVs was quantified. Thin sections from three independent cultures were surveyed and images collected of all observed MCMVs in each of these thin sections. The morphological criteria for identifying a vesicle as an MVB was based on its size being greater than 200 nm in size, and lack of dense staining compared to the neighbouring MCMV contents. While this was usually obvious (see Supplemental Figure S8), when uncertainty arose, a region‐of‐interest was drawn that included many pixels inside and outside of a candidate vesicle. If the histogram of pixel grey values showed a clear separation of two peaks, with the inside lighter than the outside, the criteria was met, and the vesicle was counted as MVB‐like. Once the mean fraction of MVBs per MCMV was established in the individual thin section, an adjustment was made based on the average size of an MVB in an MCMV (367 nm for the major axis of 50 randomly selected MVB‐like vesicles), the section thickness of 70 nm, the average size of the MCMV (average of major and minor axes of 1.08 micron), and an idealized spherical MCMV. The number of omega figures per MCMV was counted by inspection of the MCMV limiting membrane (see Supplemental Table S2).

## 3 RESULTS

### 3.1 HUVECs display a localized cluster of plasma membrane protrusions

To look for sites of EV biogenesis occurring from HUVECs using thin section EM, cells were cultured on Aclar plastic coverslips and then preserved by ultrafast freezing in liquid ethane. Frozen cells were fixed and heavy metal‐stained by freeze substitution (Walther & Ziegler, 2002), and embedded in epoxy resin. The Aclar coverslip was separated from the hardened resin, leaving the cells cleanly transferred from the coverslip to the resin, with the surface of the resin corresponding to the bottom of the cells. Seventy nanometer thick sections were cut in the *en face* orientation to cells, parallel to where the coverslip had been. As the diamond knife entered the resin, all sections were collected, including partial sections that had only grazed portions of the resin surface. Sections were collected in order so that structures spanning multiple sections could be interpreted, and distance from the surface of the coverslip in the z‐axis could be determined.

**FIGURE 1:**
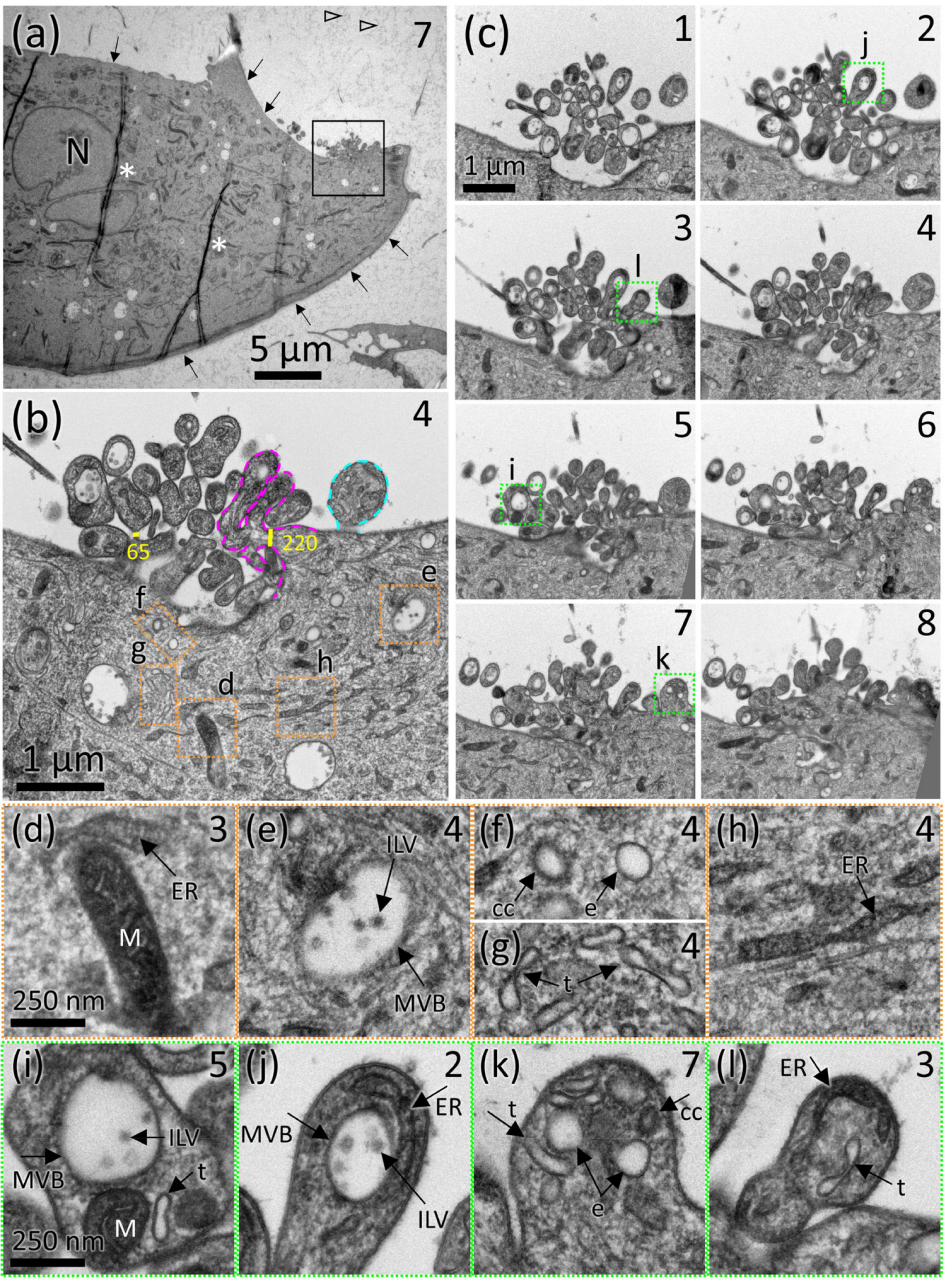
HUVECs display a localized site of membrane protrusions that contain MVBs and other membrane‐bound organelles. (a) A low‐mag transmission EM view of a portion of a HUVEC in a 70 nm‐thick section cut in the *en face* orientation, showing its elongated cell morphology and predominantly smooth plasma membrane (arrows). The number in the upper right corner indicates the number of the section in the series of serial sections shown in (c), N indicates the nucleus. Additional serial sections through this area are shown in Supplemental Movie 1. Asterisks are placed to the right of two wrinkles in the plastic section; open arrowheads point to fuzzy material that is typically observed in freeze‐substituted culture medium. (b) Boxed area in (a) from serial section 4 shows the cluster of membrane protrusions. A magenta dashed line outlines a branched protrusion; turquoise dashed line outlines an unbranched protrusion. Yellow lines indicate width in nm across protrusion necks. (c) Eight consecutive serial sections through the protrusion site with the serial section number indicted in the upper right (the greater the number, the higher above the coverslip in the z‐axis). (d‐h) Enlarged views of cytoplasmic organelles from orange boxed areas in (b) that can be identified based on their ultrastructure include: MVBs containing ILVs, mitochondria (M), endoplasmic reticulum (ER), round endosome (e), clathrin coated vesicle (cc), and tubular endosome (t). (i‐l) Enlarged views of individual protrusions from green boxed areas in (c) are shown. Arrows indicate organelles that are structurally indistinguishable from organelles identified in the cytoplasm, shown in (d‐h).

Cells were prepared at a subconfluent density, allowing isolated peripheries of individual cells where EVs might be emerging to be clearly examined. Thin sections revealed the characteristic elongated HUVEC morphology (Figure 1a). Cells displayed mostly smooth plasma membrane surfaces, however, occasionally a site spanning several micrometres of the cell surface membrane became irregularly contoured (Figure 1a, boxed area shown in 1b). Evaluation at high magnification revealed the area to be composed of a cluster of protrusions emanating from the surface of the HUVEC.

To explore the three‐dimensional organization of this ‘protrusion site’, images of the area were taken across consecutive serial sections and aligned (Supplemental Movie 1). The first eight serial sections starting from the surface of the coverslip (Figure 1c) show that the site consists of numerous protrusions, many of which were branched (Figure 1b, magenta outline). Thin necks connecting protrusions to the cell are visible in serial section 4, about 280 nm above the coverslip surface. In sections that occur below or above the level at which the protrusions connect to the cell, the protrusions cut in cross‐section appeared to be a cloud of vesicles, hovering next to the cell. For example, Figure 1c, section 1 shows the cross‐section of tips of protrusions closer to the coverslip than section 4 where they are seen connecting to the cell. At protrusion branch points and where protrusions connected to the cell, diameters often constricted to 65–200 nm wide necks (Figure 1b, yellow bars). Some protrusions spanned only three to four serial sections (280 nm in z‐height with respect to the coverslip), while others extended away from the surface of the coverslip for about 10 sections (700 nm in z‐height). Additional serial sections through this protrusion site are shown in Supplemental Movie 1. A gallery of images showing protrusion sites on other HUVECs is shown in Supplemental Figure 1. Two more examples of serial sections through protrusion sites on other HUVECs are shown in Supplemental Figures S2 and S3 and corresponding Supplemental Movies 2 and 3. These examples show protrusions that span over 1200 nm (17 sections) in height. Protrusion sites were also observed in HUVECs grown on glass and Aclar coverslips, that were prepared by conventional room temperature chemical fixation EM (Supplemental Figure S4).

To estimate the proportion of HUVECs that had a protrusion site, individual 70 nm‐thick sections from three independent cultures prepared by freeze substitution were examined, and the number of cells with and without protrusion sites were counted. Of these cells, 9% had a protrusion site (Supplemental Table S1). A model was used to estimate the mean number of protrusions per pancake‐shaped HUVEC (see methods). Based on this, it is estimated that the mean number of protrusion sites per cell is about three‐quarters. A gallery showing the range of appearances of protrusion sites in single thin sections is shown in Supplemental Figure S1.

### 3.2 Organelle‐rich protrusions contain MVBs with ILVs, endosomes, ER, and mitochondria

Organelles in the cell cytoplasm could be identified based on their stereotypical EM ultrastructure (orange boxed areas in Figure 1b, enlarged in Figure 1d‐h). In the protrusions, membrane‐bound organelles of matching structure were observed, including MVBs containing ILVs, endosomes (round, tubular, and clathrin coated), and endoplasmic reticulum (ER). Occasionally, mitochondria, recognizable by their distinct cristae, double membranes and dark electron dense staining were also observed in the protrusions (green boxed areas in Figure 1c, enlarged in Figure 1i‐l).

### 3.3 Vesicles were present on the coverslip surface that were not connected to cells and contained MVBs and other membrane‐bound compartments

Based on the protrusion morphology—thin necks connecting protrusions to the cell and at branch points—it was hypothesized that the protrusion site is a specialized site for the assembly and budding of EVs. Because the membrane would be derived from the HUVEC plasma membrane, such an EV would qualify as a microvesicle. Furthermore, should a protrusion containing membrane‐bound organelles pinch off and diffuse into the extracellular milieu, it would create an microvesicle containing multiple compartments, i.e., a multi‐compartmented microvesicle (MCMV). If so, diffusing MCMVs may occasionally contact and stick to the coverslip, and remain attached during preparation for EM. To explore this possibility, areas beyond cell peripheries on seemingly empty expanses of coverslip were searched in the *en face* sections that were closest to the coverslip surface (illustrated in Supplemental Figure S5 and corresponding Supplemental Movie 4).

**FIGURE 2:**
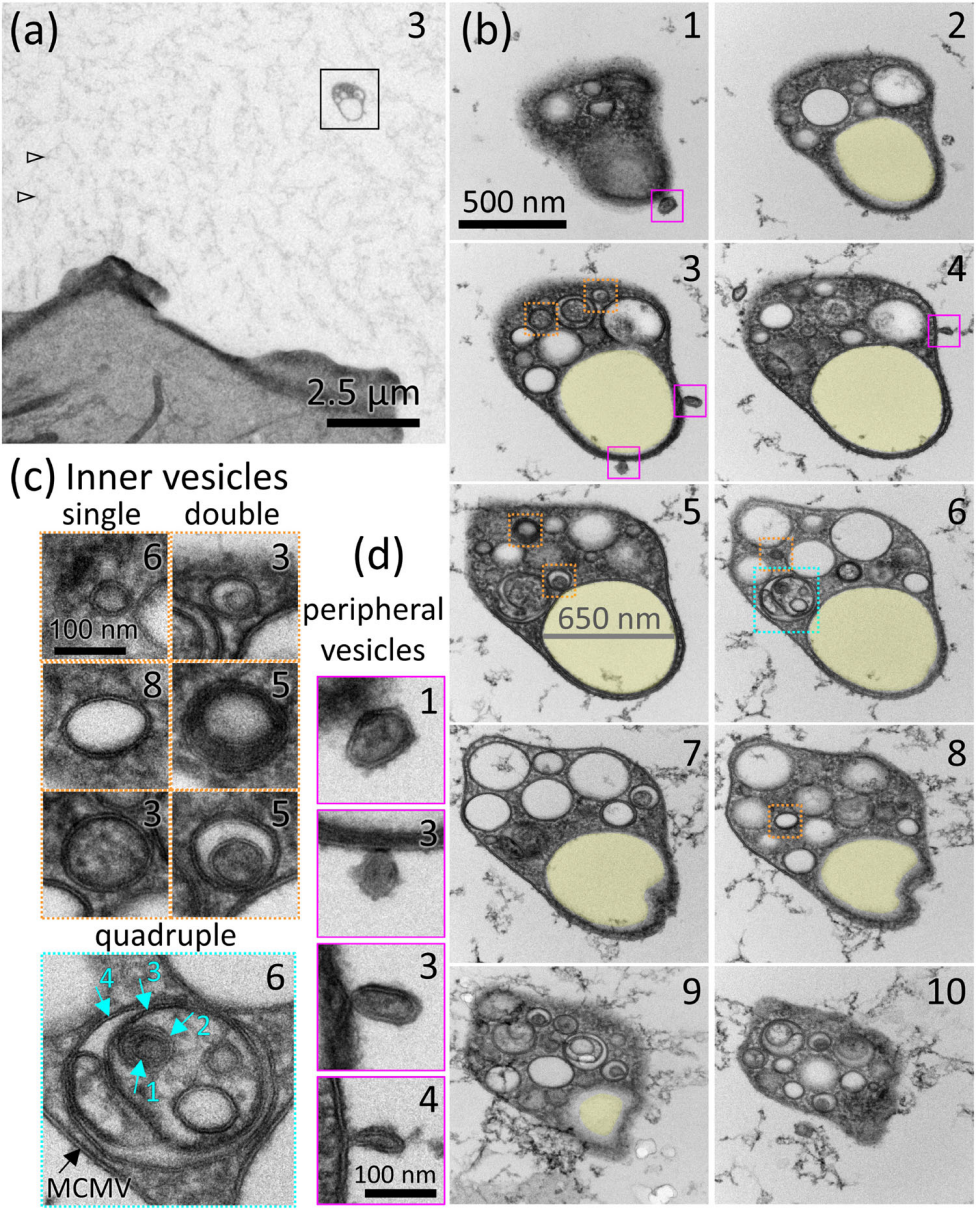
**MCMVs are found on the coverslip surface and are completely independent from neighbouring cells**. (a) A low‐magnification view of a presumptive MCMV (boxed) located 7 μm beyond the periphery of the nearest cell. Arrows point to fuzzy material that is typically observed in freeze‐substituted culture medium. The complete series of serial sections through the MCMV boxed in (a) is shown in (b) and Supplemental Movie 5. The number in the upper right corner indicates the number of the serial section (ascending number indicates moving higher in the z‐axis with respect to the surface of the coverslip). Examples of vesicles inside the MCMV are boxed in orange or turquoise and shown in (c). A 650‐nm diameter MVB‐like vesicle that spans almost the entire series of sections but is devoid of ILVs is shaded yellow in (b). (c) Examples of MCMV inner vesicles that are single vesicles or double vesicles, boxed orange, or a quadruple vesicle, boxed in turquoise. Turquoise arrows indicate four layers of membrane of the quadruple vesicle, inside the MCMV limiting membrane (black arrow), amounting to five membrane layers. Inner vesicles displayed various combinations of dark, light, or granular lumens. (d) Small vesicles associated with the outer periphery of the MCMV are boxed in magenta in (b) and shown enlarged in (d).

In such areas, presumptive MCMVs were found beyond the periphery of cells. An example shown in Figure 2a was located about 7 μm from the nearest cell. A complete set of 10 serial sections through this MCMV was obtained starting at from the very first section that contained the MCMV within the first 70 nm of the surface of the coverslip, to the 10^th^ and last section in which the MCMV appeared (Figure 2b and Supplemental Movie 5). Examination of the serial sections confirmed that the MCMV was not connected to a neighbouring cell by a membrane extension, nor were remnants of detached membrane connections observed.

### 3.4 MCMVs contain MVBs with ILVs and other membrane‐bound compartments

Serial sections through MCMVs demonstrated that they contained round and tubular membrane‐bound compartments in addition to a dense cytoplasm (Figure 2b). The vesicular compartments varied in diameter from 45 to 650 nm. Lumens of the vesicles below 200 nm in diameter varied from electron lucent to electron dense with a smooth or granular texture (Figure 2c). The larger compartments were most often electron lucent, like the lumens of MVBs (Figure 1b,e,i,j). Often, an MCMV inner vesicle contained another vesicle, that is, a vesicle inside a vesicle, or double vesicle (Figure 2c). The lumens of the double vesicles often differed in texture or electron density from the vesicle in which they were enclosed (see Figure 2c, double vesicles from section 5). On occasion, MCMV inner vesicles contained multiple vesicles. A quadruple vesicle in Figure 2c from section 6 shows an inner vesicle that contains a round 250 nm vesicle and a 60 nm thick tubule, both with granular textured lumens. The 250 nm round vesicle contains four additional vesicles with diameters ranging from 50 to 75 nm with smooth light to dark grey lumens. One of these vesicles contains a sixth, 45 nm vesicle, also with a smooth grey lumen. In this configuration, there are five layers of membrane between the contents of the sixth vesicle and the extra‐MCMV milieu.

Also present in the MCMV shown in Figure 2b, is a 650 nm sub‐compartment (shaded yellow), along with some ∼300 nm compartments that are similar in roundness and electron lucency to MVBs, but they do not contain any ILVs. The presence of putative empty MVBs inside MCMVs raised the possibility that MVBs might be capable of releasing their ILVs (to become exosomes) from an MCMV after it has transited away from its parent cell. This observation provided circumstantial evidence for ILV secretion (exosome release) from MCMVs.

### 3.5 MVBs appear to fuse with MCMV membrane and release exosomes

**FIGURE 3:**
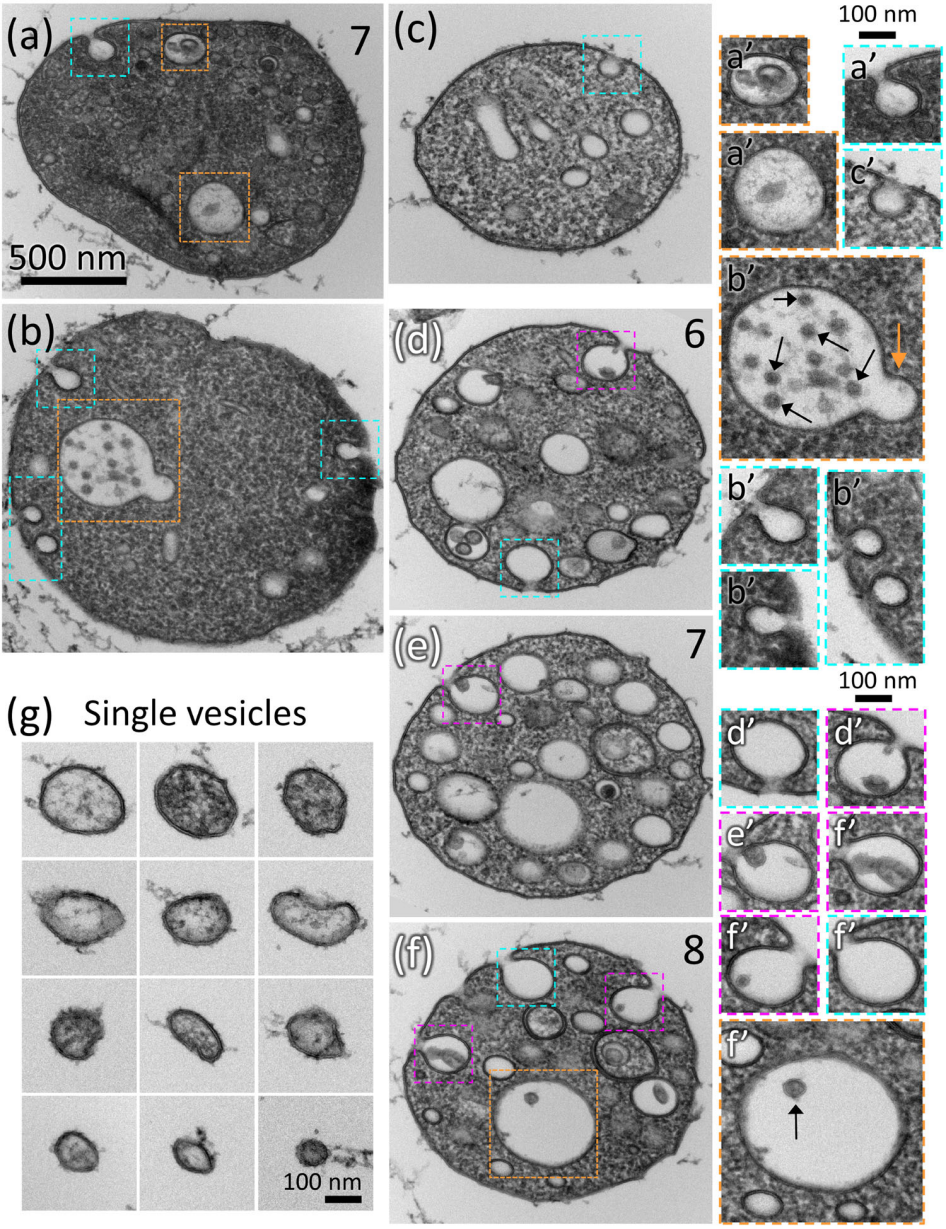
**MCMVs contain MVBs that fuse with the MCMV membrane to release exosomes**. MCMVs containing organelles that are structurally similar or identical to MVBs are boxed orange in (a), (b), and (f) and shown to the right enlarged in orange boxes, (a’), (b’), and (f’). The MVB in (b’) appears to undergo an outward budding remodelling step (orange arrow). Black arrows indicate ILVs in (b’) and (f’). (a‐c) MCMVs with one or more omega‐figures on their limiting membrane that do not contain ILVs (boxed turquoise and enlarged to the right in turquoise boxes (a’), (b’), and (c’). (d‐f) Three serial sections through an MCMV (section number indicated in upper right corner) in which omega figures occur in each section. Some omega figures contain ILVs (magenta boxes, enlarged to the right in (d’), (e’), and (f’)) and some do not contain ILVs (turquoise boxes, enlarged to the right in (d’) and (f’)). (g) Single‐membrane vesicles located on the coverslip surface that are structurally like the inner vesicles of MCMVs. Scalebar in (a) applies to (a‐f). Supplemental Figure 6 and Movie 6 show serial sections of the MCMV shown in (a). Supplemental Figure 7 and Movie 7 shows additional serial sections of the MCMV shown in (d‐f).

If exosomes are released from MCMVs, MVBs containing ILVs must be observed inside MCMVs, and evidence of membrane fusion between the MVB the MCMV membrane must be detected. Sections of MCMVs were examined (Figure 3), and examples of MVB‐like vesicles, indistinguishable from MVBs observed in cell cytoplasm were observed (boxed orange in Figure 3b,f and 3b’,3f’). The MVB in Figure 3b’ appears to be undergoing an outward budding event (orange arrow), suggesting active remodelling of MVBs inside MCMVs. Other MVB‐like vesicles inside MCMVs were observed (boxed orange in Figure 3a and 3a’, and see Supplemental Figure S6). On average, about one MVB‐like vesicle per MCMV was detected (Supplemental Table S2).

The most stringent structural test for membrane fusion between organelles and the plasma membrane is the omega‐shaped figure, describing the shape of a fusion intermediate in a cross‐section thinner than the fusion pore (Douglas, 1973). Thus, if MCMVs secrete exosomes, omega figures of fusing MVBs should occasionally be visible on the MCMV limiting membrane, especially since the cultures were preserved by ultrafast freezing which occurs in milliseconds and can capture dynamic events like exocytosis.

Further examination revealed about 43% of MCMVs had one or more omega‐shaped profiles on their limiting membrane (Supplemental Table S2 and turquoise boxes in Figure 3a‐d,f and a’,b’,c’,d’,f’). These profiles resembled smaller versions of the exocytic profiles of MVBs fusing with the cell plasmalemma in reticulocytes that also preserved by ultra‐fast freezing and processed by freeze substitution and thin section EM (Harding et al., 1983).

If exosomes are released from MCMVs via these omega profiles, ILV‐like vesicles might be seen inside or nearby the fusion event. Indeed, some of the omega figures contained ∼50–100 nm vesicles with grey lumens that were indistinguishable from ILVs (magenta boxes in Figure 3d‐f and d’‐f’). Omega figures of fusion can span two sections and may appear empty in one section but seen to contain an ILV in the neighbouring section (Supplemental Figures S6 and S7, and Supplemental Movies 6 and 7). About 65% of the MVB‐like vesicles had ILV‐like vesicles within them (Supplemental Table S2). A gallery showing examples of MCMVs preserved by freeze substitution are shown in Supplemental Figure S8, and MCMVs preserved by conventional room temperature chemical fixation are shown in Supplemental Figure S4.

### 3.6 Single‐membrane vesicles corresponding to MCMV's internal vesicles are found on coverslip surface

If MCMVs release some of their internal vesicles, including ILVs (exosomes), they too might attach to the coverslip surface, and it might be possible to find single‐membrane vesicles that match the size and appearance of MCMV inner vesicles in the first one or two sections cut along the surface of the coverslip. To test the prediction of this hypothesis, such areas were searched at high magnification, and single‐membrane vesicles ranging in size from ∼50 to 200 nm were found (Figure 3g). Like the vesicles observed inside MCMVs, the vesicles had grey smooth or granular interiors. The observation of these vesicles was consistent with their release from MCMVs.

## 4 DISCUSSION

**FIGURE 4:**
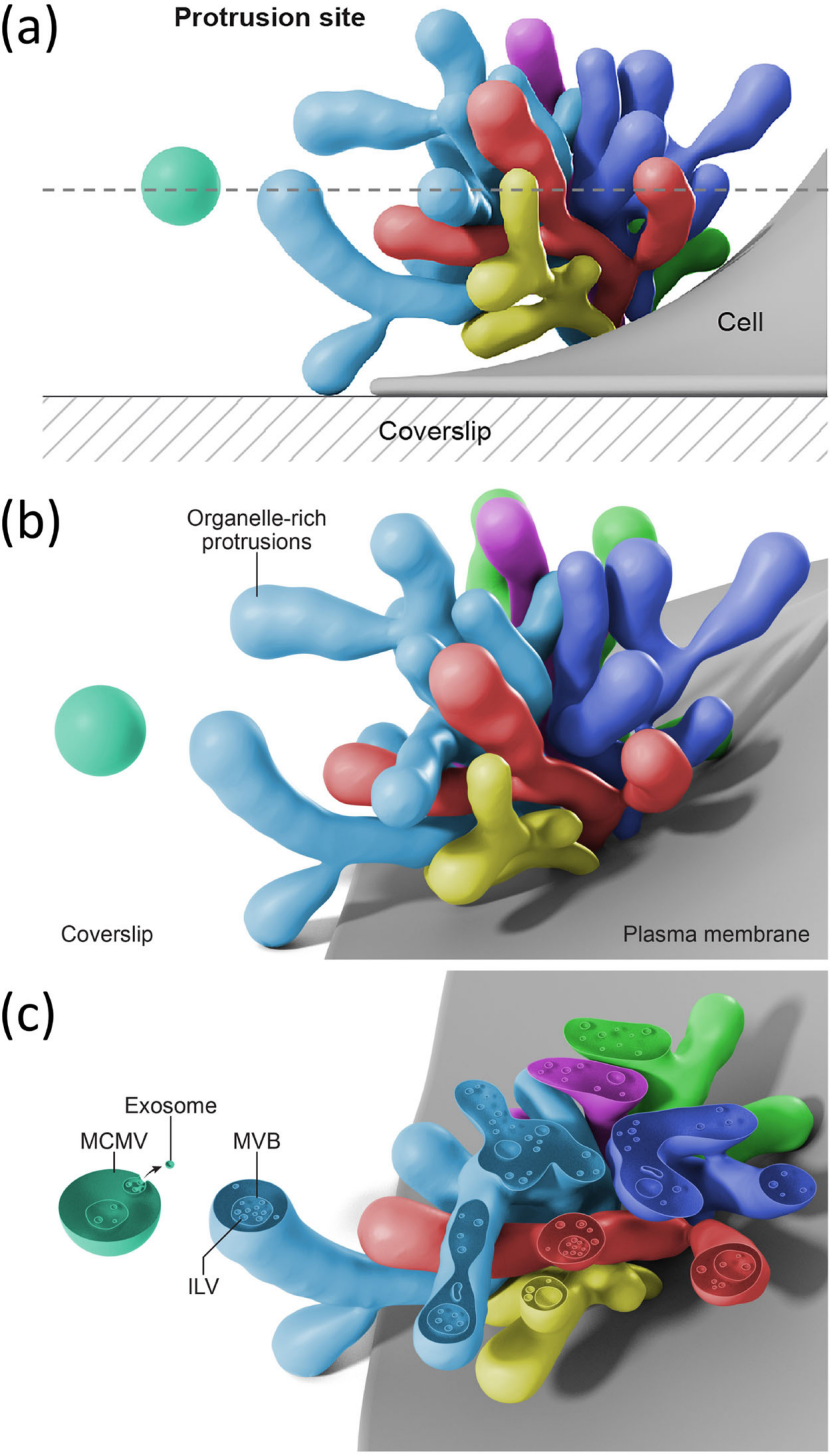
Artistic representation based on data sets of a protrusion site on a cultured endothelial cell and omega figures on a MCMV a distance from a cell. (a) Depiction of several protrusions clustered on a cultured endothelial cell surface, and a nearby pinched off MCMV floating in the extracellular milieu. A slice plane in the *en face* orientation relative to the coverslip is represented by the dashed line. (b) A view at a slightly different angle shows that the protrusions are often branching. (c) A slice through the protrusions and the MCMV shows internal round and tubular vesicular organelles, including MVBs containing ILVs, inside the protrusions and the MCMV. An omega figure on the MCMV limiting membrane indicates exosome secretion from the MCMV.

Here a new class of microvesicle is described, termed MCMV, that buds from cellular protrusions clustered on the plasma membrane of HUVECs (Illustrated in Figure 4). The cellular membrane protrusions contain vesicular cargo that, when compared with cytoplasmic organelles, could be identified as MVBs with ILVs, endosomes (round, tubular and clathrin coated), ER, and mitochondria. Serial sections showed that the protrusions are a few hundred nanometers to 1 micron thick and in some cases extended up in the Z axis relative to the coverslips for more than ∼1200 nm, beyond the scope of the serial sections analyzed. Protrusions were often branched and intermingled. At branch points and connections to the cell, the protrusions often became constricted to thin necks of 65–200 nm (yellow bars in Figure 1b). Exploration of the coverslip surface between cells revealed MCMVs that were immobilized on the coverslip and contained MVB‐like vesicles containing ILV‐like vesicles, in addition to other vesicle types. The direct observation of omega figures joining membranes of internal vesicles of MCMVs with the peripheral membrane of MCMVs, and the many images of ILV‐like vesicles in and immediately adjacent to the fusion pore of omega figures, and associated with the periphery of MCMVs (Figure 2d), together suggest to us that ILVs can be released from MCMVs—a function akin to the exocytosis of ILVs from cells. Since that could only be conceivable if MCMVs contain compartments akin to MVBs of cells, such a hypothesis would be unique to MCMVs. Preservation, by fast‐freezing, of omega figures on the MCMV‐limiting membrane showed a lack of any membrane coat. Thus, these omega figures are not the result of any coat‐mediated endocytosis such as those mediated by clathrin or caveolin. It also seems of low probability, but not impossible, that ILV‐sized vesicles are captured and internalized into MCMVs from the relatively vast volume of culture medium. Taken together, these observations suggest a novel pathway by which a subset of exosomes are released from a transiting MCMV after pinching off from a protrusion on the HUVEC surface.

A previous scanning and transmission EM study of HUVECs described what is likely the same site of protrusions as we document and reported that vesicles shed from this specialized site on the plasma membrane contain proteases that promote angiogenesis (Taraboletti et al., 2002). Similarly, a separate scanning EM study showed discrete areas of membrane protrusions on otherwise relatively smooth surface membranes of unstimulated HUVECs (Combes et al., 1999). Indeed, various specialized domains of membrane protrusions have been shown to shed microvesicles on a number of cell types (Rilla, 2021).

The rapid freezing, freeze substitution and serial section EM methodology utilized here (Walther & Ziegler, 2002) is currently being applied to other cell types to determine if MCMVs are released by other cell types, or are unique to HUVECs. A growing number of cryo‐EM studies document EVs containing internal vesicles derived from diverse sources such as human plasma, cerebrospinal fluid, urine, and ejaculate, a human mast cell line, and rodent neuronal culture media suggesting that release of MCMVs is a mechanism conserved in other cell types (Arraud et al., 2014; Brisson et al., 2017; Emelyanov et al., 2020; Gamez‐Valero et al., 2019; Hoog & Lotvall, 2015; Matthies et al., 2020; Zabeo et al., 2017). Similarly, EVs containing vesicles have been documented in some thin section EM studies of chemically fixed cells (Valcz et al., 2019).

A challenge in the study of EVs has been the isolation of EV subpopulations. Though possessing different sites of origin, microvesicles and exosomes share overlapping size ranges, molecular compositions, and densities, rendering biochemical enrichment and characterization of EV subsets a challenge (Kowal et al., 2016). The findings of this paper suggest that part of the difficulty may arise from EVs that consist of exosomes inside of microvesicles. Their presence could go unrecognized or misinterpreted as apparent overlap in biophysical properties. Moreover, attempts to separate microvesicles from exosomes may prove futile when MCMVs are both.

Other cellular processes produce EVs containing internal vesicle compartments, similar to MCMVs. For example, cells undergoing apoptosis fragment into apoptotic bodies containing micronuclei and other organelles, and range in size from ∼500 nm to several microns in diameter (Xu et al., 2019). However, none of the MCMVs described in the current paper appeared to contain micronuclei. In addition, cultures screened for cytotoxicity showed minimal cell death (estimated at 1 dead cell per 6000 cells surveyed). A second class of EV that contains internal compartments is exophers, large (∼4 μm diameter) membrane‐bound organelles which are extruded from touch neurons in *Caenorhabditis elegans* (Melentijevic et al., 2017) and murine cardiomyocytes (Nicolas‐Avila et al., 2020) to expel potentially toxic materials such as dysfunctional mitochondria and protein aggregates. Additionally, in *C. elegans*, exophers released by muscle cells in pregnant females transfer nourishing yolk proteins to support developing embryos (Turek et al., 2021). Exophers are the largest class of EV (up to 15 μm), and characteristically contain numerous mitochondria, which is not a distinguishing feature of MCMVs (Turek et al., 2021). There can also be a tube connecting the exopher to the cell from which it was extruded, but no connections were observed between MCMVs and cells.

Migrasomes are a third type of EV that contains numerous internal vesicles, and they perhaps share the most similarities with MCMVs, however migrasomes were shown not to contain MVBs (Ma et al., 2015). Migrasomes form by a migration‐dependent mechanism at the termini and branch points of retraction fibres emanating from the trailing edge of migrating cells such as NRK cells (Ma et al., 2015) and other cell types and tissues (Di Daniele et al., 2022). However, MCMVs were not attached to retraction fibres, nor did they have 'tails' of broken fibres as seen in electron micrographs of migrasomes (Jiao et al., 2021; Ma et al., 2015; Yu & Yu, 2021). Also, remnants of retraction fibres were not observed on the coverslip in abundance, and the distribution of MCMVs on the coverslip surface appeared random relative to neighbouring cells and were not concentrated on one side of a cell (potentially the trailing edge). Since the protrusions on HUVECs extend away from the coverslip, rather than adhering to the coverslip substrate like retraction fibres, our interpretation is that MCMVs pinch off and diffuse in the culture medium before occasionally landing and becoming immobilized on the coverslip surface. It will be interesting to confirm this interpretation in future studies and determine if the location of the protrusion site on HUVECs correlates with the trajectory of cell movement.

Of note, the HUVECs cultured in vitro used in this study differ in some ways from tissue vascular endothelium, and future studies are needed to explore these findings in more physiological system. The glycocalyx of HUVECs may differ from those in tissues (Chappell et al., 2009), and an ILV could have more trouble traversing the glycocalyx of a tissue cell once released. Also, the vascular endothelium in tissue is not proliferating, whereas HUVECs in culture are exposed to several angiogenic growth factors in the medium and replicate many features of endothelial cells undergoing an angiogenic response. Angiogenic responses are known to alter the production and contents of endothelial‐derived EVs (Alfi et al., 2021), and EVs produced by endothelial cells can regulate angiogenic responses (Chen et al., 2018; Lamichhane et al., 2017). The EV production studied here may be relevant to their role in angiogenesis. Furthermore, the HUVECs analyzed were subconfluent to better observe potential sites of EV biogenesis on the cell peripheries—possibly release of MCMVs occurs in response to a wound‐like state to influence wound healing. Future studies are needed to determine if MCMVs are released from confluent endothelial cells in in vivo and in vitro.

## 5 CONCLUSION

In summary, we have described a domain of protrusions extending from the plasma membrane of HUVECs that contain membrane bound organelles including MVBs. MCMVs bud from the protrusions and contain vesicular compartments, including MVBs that can fuse with the MCMV limiting membrane and release exosomes. This implies that the function of MCMVs is signalling rather than removal of cellular material, as has been proposed for exophers and migrasomes. To be functional as signalling entities, EVs must deliver messages, in the form of bioactive molecules to recipient cells. Packaging cargos inside multiple layers of membrane rather than a unilamellar carrier could shield EV contents from degradation in the extracellular space, enabling them to voyage farther before being released from the MCMV or taken up into recipient cells. Multiple layers of membrane could also help vesicle contents avoid lysosomal degradation in the recipient cytoplasm and/or reach the nucleus. Additionally, grouping multiple vesicles of related signalling molecules into a single EV could deliver contents as a functional unit, rather than relying on coincidental arrival of components in separate EVs, at the right place and in the right quantities allowing for more efficient signalling. MCMVs can be evaluated as a new type of organelle‐containing microvesicle, and a potential source of exosome release that occurs remotely from the parent cell, adding new considerations to when, where, and how EVs are assembled and released from the endothelium and potentially other cells and tissues.

## AUTHOR CONTRIBUTIONS

**Jennifer D. Petersen**: Conceptualization; Formal analysis; Investigation; Methodology; Validation; Visualization; Writing – original draft. **Elena Mekhedov**: Conceptualization; Investigation; Methodology; Writing – review & editing. **Sukhbir Kaur**: Methodology; Writing – review & editing. **David D. Roberts**: Funding acquisition; Methodology; Resources; Supervision; Writing – review & editing. **Joshua Zimmerberg**: Conceptualization; Funding acquisition; Methodology; Project administration; Resources; Supervision; Validation; Writing – review & editing

## CONFLICT OF INTEREST STATEMENT

The authors declare no conflict of interest.

## Supporting information


**Supplemental Table 1**: Frequency of protrusion sites on HUVECs.
**Supplemental Table 2**: The proportion of MCMVs in thin sections.
**Supplemental Figure 1**: Gallery of protrusion sites on HUVECs.
**Supplemental Figure 2**: Example of serial sections of a protrusion site and contents thereof.
**Supplemental Figure 3**: Example of serial sections of a protrusion site.
**Supplemental Figure 4**: Protrusion sites and MCMVs preserved by room temperature chemical fixation.
**Supplemental Figure 5**: Illustration of en face serial sections closest to cover slip.
**Supplemental Figure 6**: Additional sections through MCMV showing MVB‐like structures.
**Supplemental Figure 7**: Additional sections through MCMV showing omega figures with internal ILV.
**Supplemental Figure 8**: Gallery of MCMVs preserved by freeze substitution.

Supplemental Movies

## Data Availability

Data is available upon request from the corresponding author.
