# Supplementary material for: Endothelial cells release microvesicles that harbour multivesicular bodies and secrete exosomes
Source: J Extracell Biol. 2023 Mar 30;2(4):e79. doi: 10.1002/jex2.79 (PMC11080864; doi:10.1002/jex2.79)
Supplement: Supplementary file 1 — Supplemental Table 1: Frequency of protrusion sites on HUVECs. Supplemental Table 2: The proportion of MCMVs in thin sections. Supplemental Figure 1: Gallery of protrusion sites on HUVECs. Supplemental Figure 2: Example of serial sections of a protrusion site and contents thereof. Supplemental Figure 3: Example of serial sections of a protrusion site. Supplemental Figure 4: Protrusion sites and MCMVs preserved by room temperature chemical fixation. Supplemental Figure 5: Illustration of en face serial sections closest to cover slip. Supplemental Figure 6: Additional sections through MCMV showing MVB‐like structures. Supplemental Figure 7: Additional sections through MCMV showing omega figures with internal ILV. Supplemental Figure 8: Gallery of MCMVs preserved by freeze substitution. [file JEX2-2-e79-s001.docx]

**Endothelial cells release microvesicles that harbor multivesicular bodies and secrete exosomes**

Jennifer D. Petersen^1^, Elena Mekhedov^1^, Sukhbir Kaur^2^, David D. Roberts^2^, Joshua Zimmerberg^1*^

^1^Section on Integrative Biophysics, Division of Basic and Translational Biophysics, *Eunice Kennedy Shriver* National Institute of Child Health and Human Development, National Institutes of Health, Bethesda, USA 20892

^2^Laboratory of Pathology, Center for Cancer Research, National Cancer Institute, National Institutes of Health, Bethesda, USA 20892

*Corresponding author

**SUPPORTING INFORMATION**

**SUPPLEMENTAL FIGURES and Tables:**

SUPPLEMENTAL TABLE 1

SUPPLEMENTAL TABLE 2

SUPPLEMENTAL FIGURE 1

SUPPLEMENTAL FIGURE 2

SUPPLEMENTAL FIGURE 3

SUPPLEMENTAL FIGURE 4

SUPPLEMENTAL FIGURE 5

SUPPLEMENTAL FIGURE 6

SUPPLEMENTAL FIGURE 7

SUPPLEMENTAL FIGURE 8

**SUPPLEMENTAL MOVIES:**

SUPPLEMENTAL MOVE 1

SUPPLEMENTAL MOVIE 2

SUPPLEMENTAL MOVIE 3

SUPPLEMENTAL MOVIE 4

SUPPLEMENTAL MOVIE 5

SUPPLEMENTAL MOVIE 6

SUPPLEMENTAL MOVIE 7


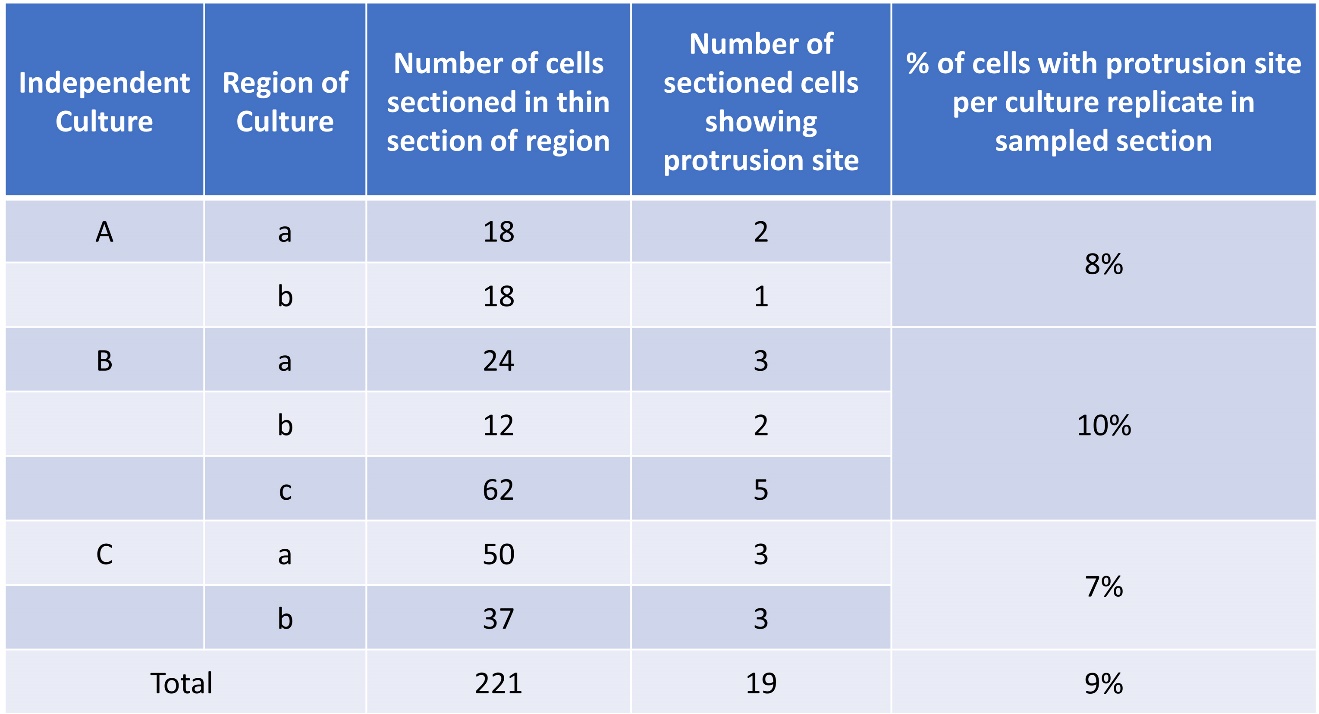


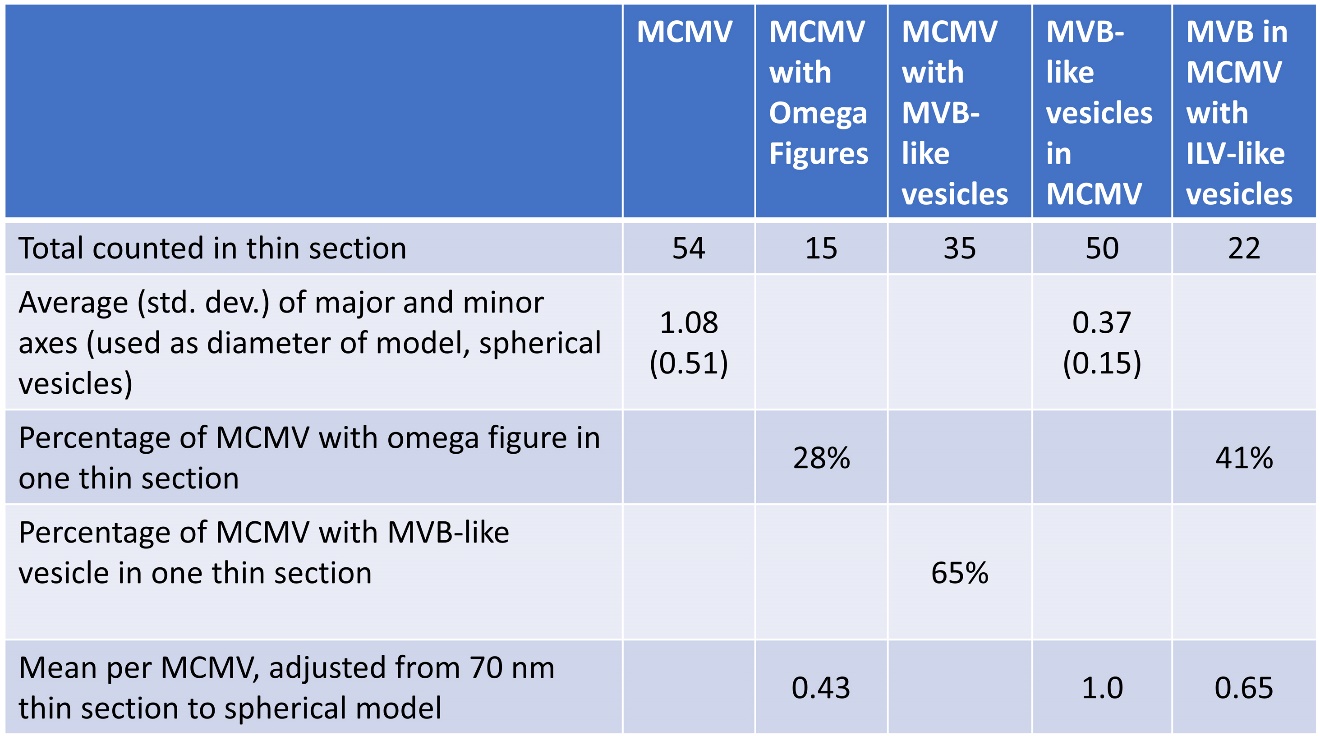
Supplemental Table 1: Frequency of protrusion sites on HUVECs. The number of cells with and without protrusion sites, in thin sections from at least two different culture regions, in three independent cultures are summarized.

Supplemental Table 2: The proportion of MCMVs in thin sections from three independent HUVEC cultures that contain MVB-like vesicles, with and without ILV-like vesicles, and omega figures on their peripheral membranes is summarized.


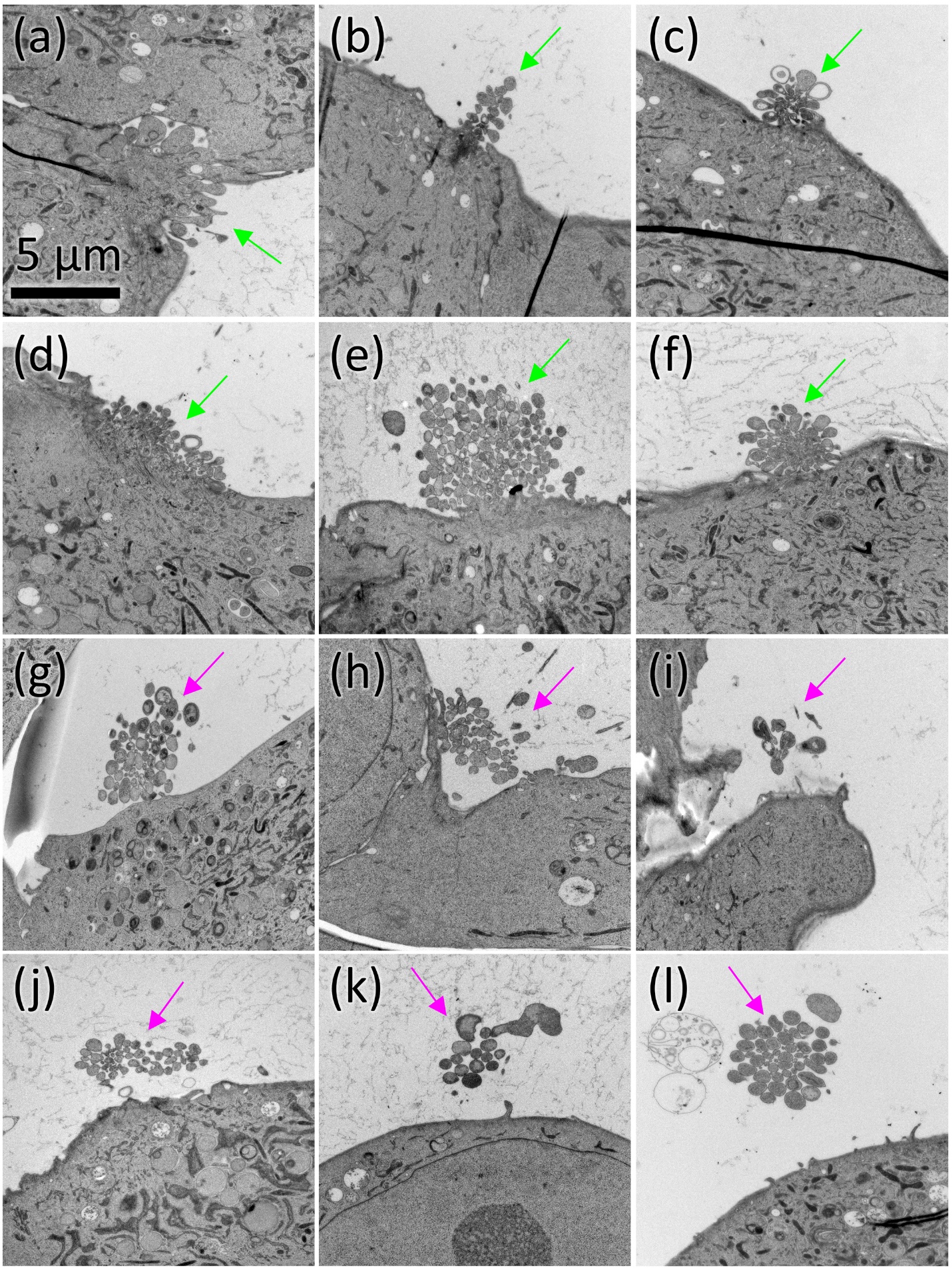


**SUPPLEMENTAL FIGURE 1:** Gallery of protrusion sites on HUVECs in single thin sections of cells prepared by freeze substitution. (a-f) protrusion sites (green arrows) in which the plane of section passes through protrusion necks, showing connections to cells. (g-l) protrusion sites (magenta arrows) in which the plane of section occurs above or below the protrusion necks, such that protrusions are cut in cross-section and connections to cells are not visible.

**
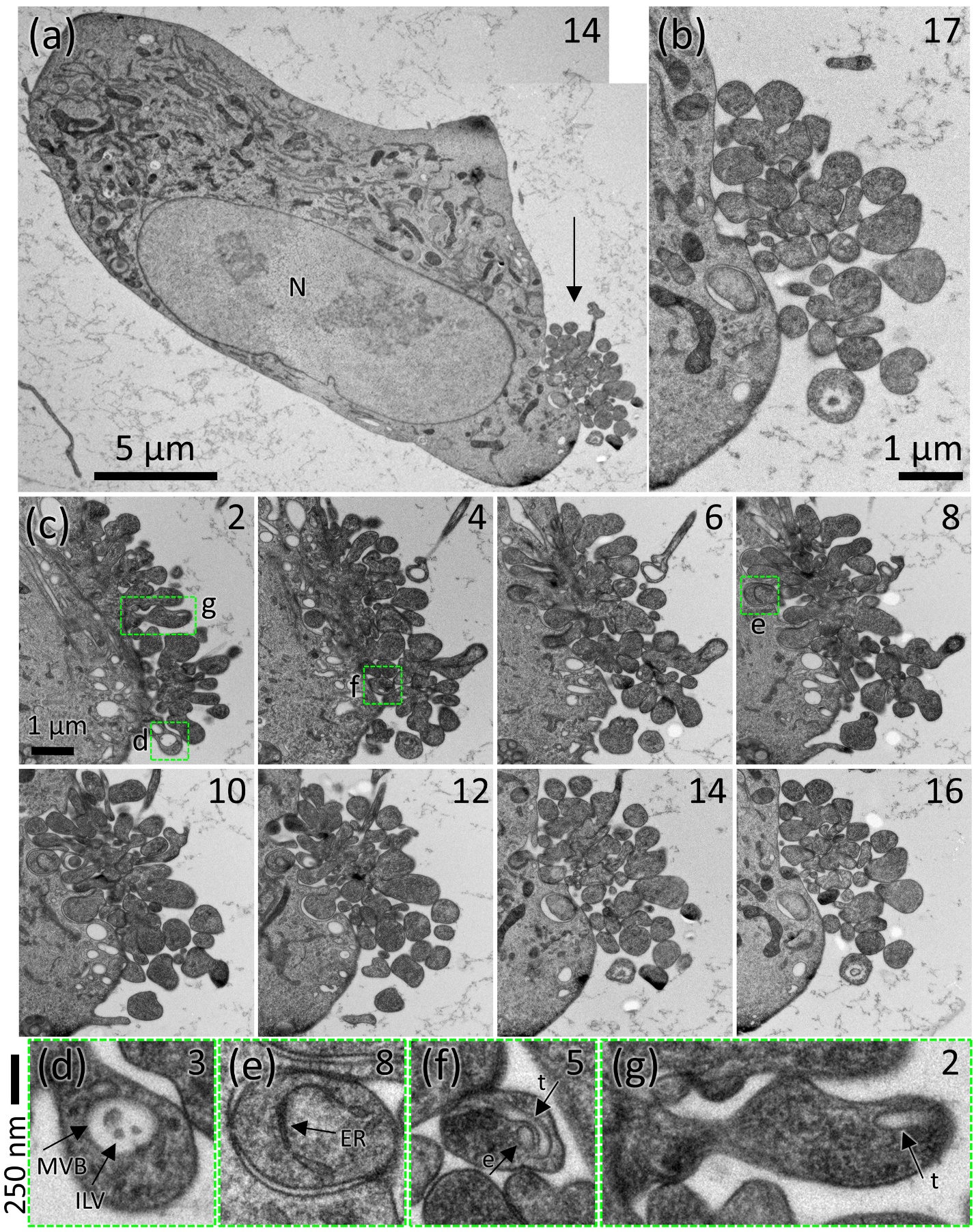
**

**SUPPLEMENTAL FIGURE 2:** (a) A thin section through an entire HUVEC shows a smooth cell surface interrupted by site of protrusions localized to one end of the cell (arrow). Serial section number is indicated in the upper right corner of each image. The higher the number, the greater the distance from the coverslip. N, nucleus. (b) Enlarged view of the protrusion site as it appears in section 17, ~1200 nm above the surface of the coverslip. At this height, the protrusions are cut in cross-section and do not appear attached to the cell. (c) Even numbered sections through the protrusion site are shown with section number in the upper right corner. All sections can be viewed in Supplemental Movie 2. Green boxed areas show protrusions that are enlarged in (d-g) with arrows indicating membrane-bound organelles including MVBs containing ILVs, endoplasmic reticulum (ER), round endosome (e), and tubular endosome (t).

**
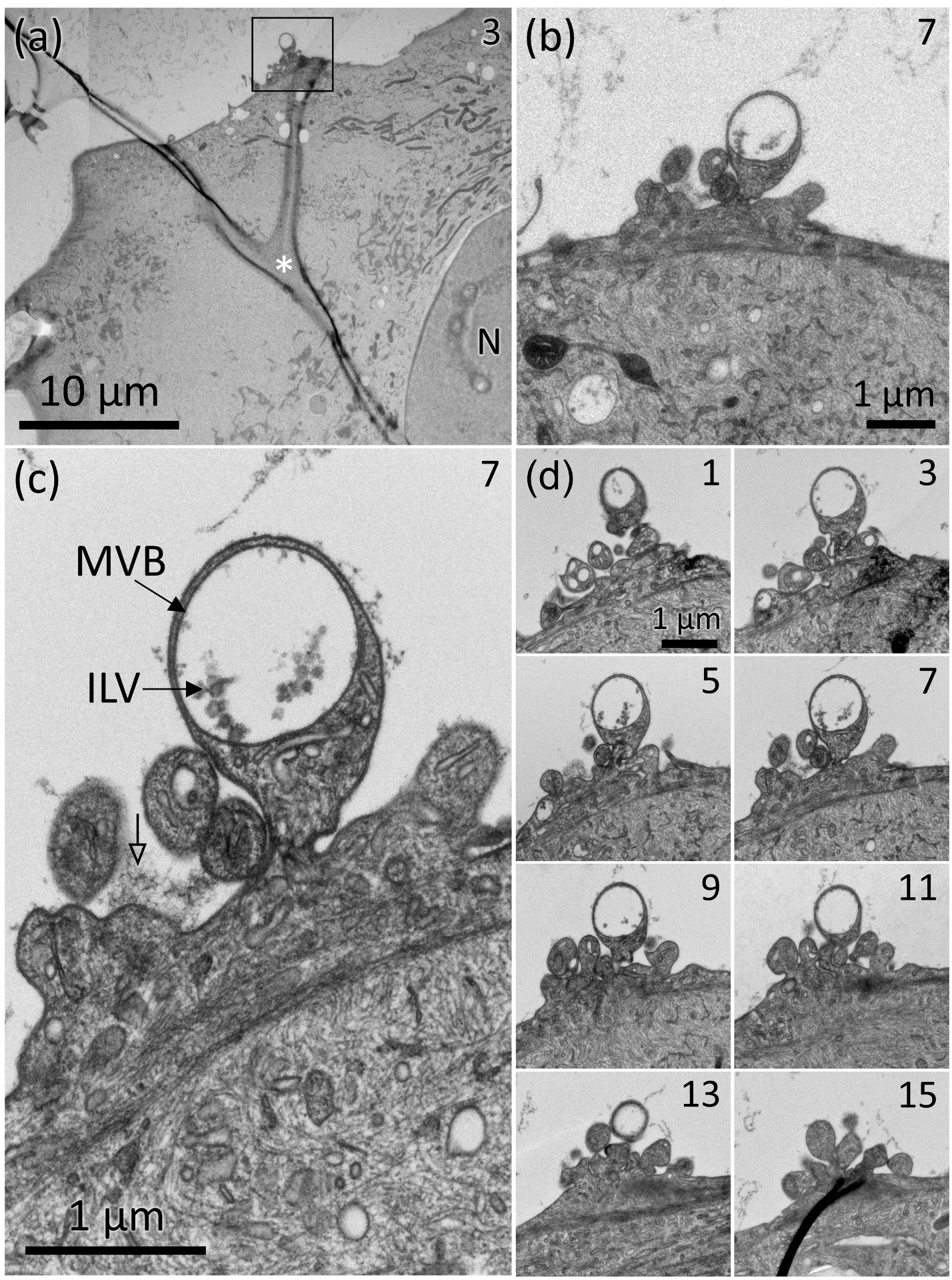
**

**SUPPLEMENTAL FIGURE 3:** (a) A site of protrusions (boxed) on the otherwise smooth surface of a HUVEC. White asterisk indicates a Y-shaped wrinkle in the section. N, nucleus. Serial section number is indicated in the upper right corner of each image. An enlarged view of the protrusion site boxed in (a) is shown in (b) and (c). Arrows indicate an MVB containing ILVs in a prominent bulb-shaped protrusion. Open arrowhead indicates fibrous material occasionally present between protrusions. (d) Odd-numbered serial sections through the protrusion site are shown, see also Supplemental Movie 3.


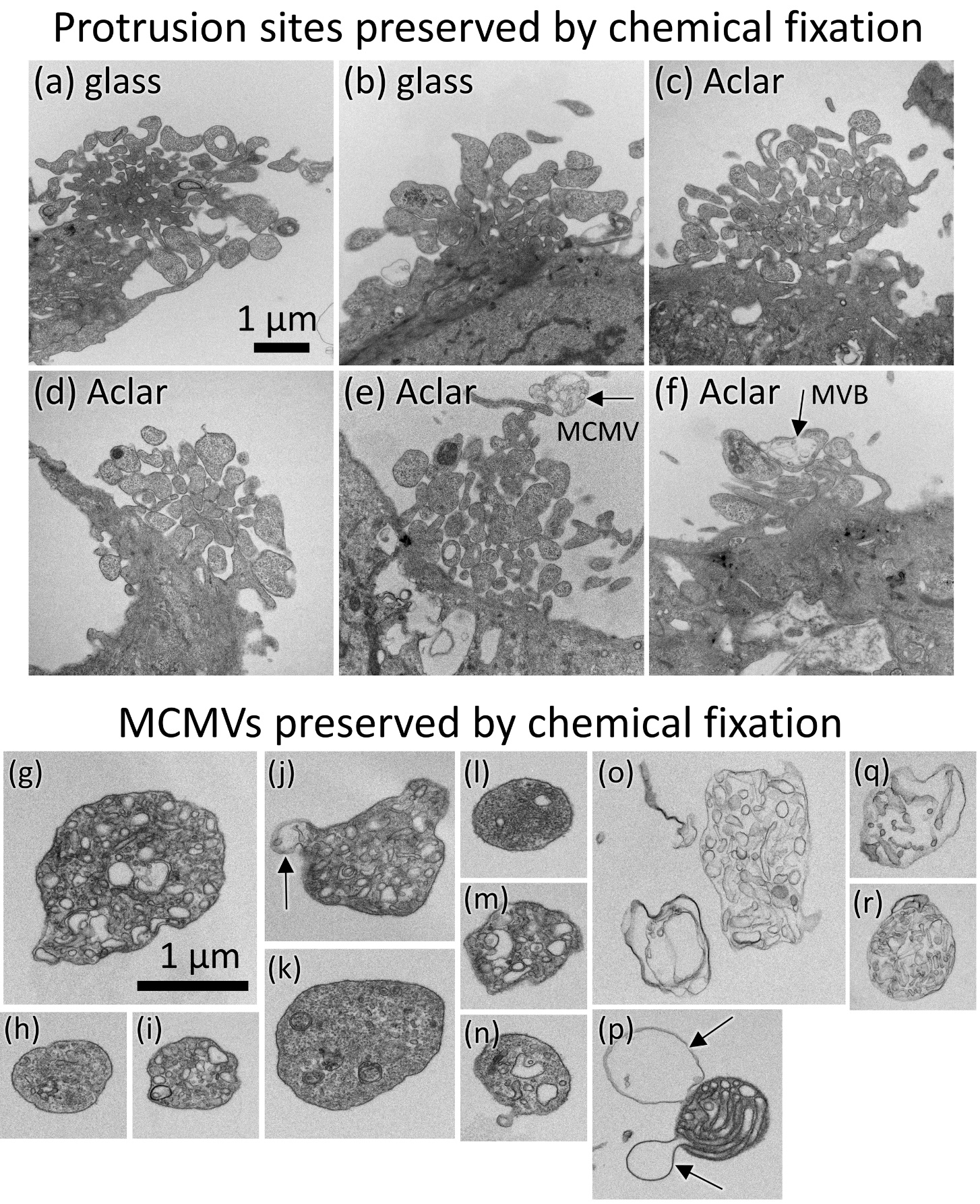


**SUPPLEMENTAL FIGURE 4:** Protrusion sites and MCMVs preserved by room temperature chemical fixation. (a-b) Protrusion sites on HUVECs cultured on glass coverslips. (c-f) Protrusion sites on HUVECs cultured on Aclar coverslips. Arrow in (e) indicates an MCMV near the site of protrusions. Arrow in (f) indicates an MVB in a protrusion. (g-r) MCMVs preserved by chemical fixation. Some MCMVs appear to have contents extracted (o-r) and/or membrane blebs (arrows in (p) and (j)) which may be artifacts of chemical fixation.

**
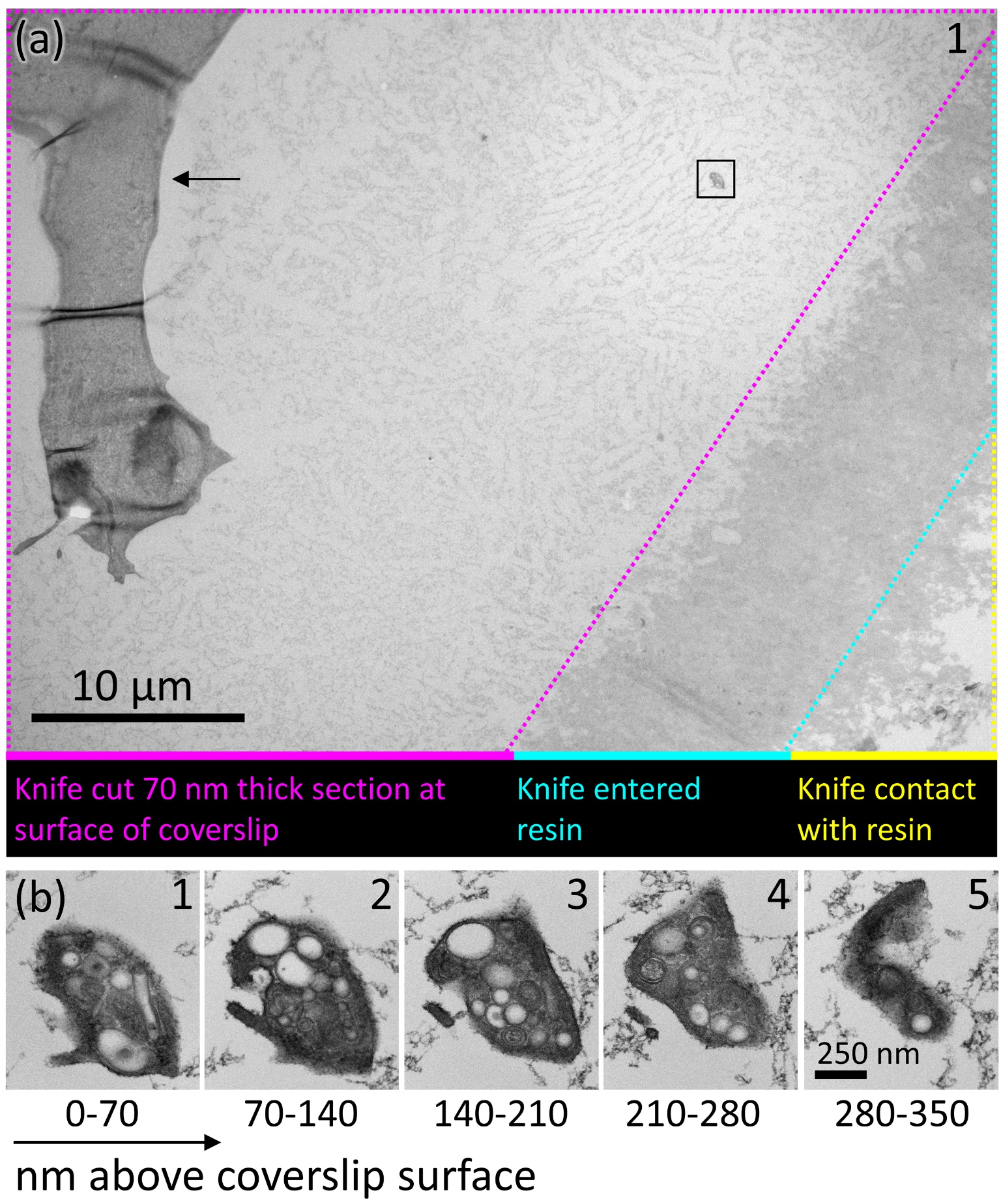
**

**SUPPLEMENTAL FIGURE 5:** (a) Field of view showing an MCMV (boxed) located 25 µm from the nearest cell (arrow) and 10 µm from where the knife entered the resin during sectioning. The ragged edge (indicated by yellow boundary) is where the knife contacted the surface of the resin, then entered the resin (turquoise boundary), and then began cutting a 70 nm thick section (magenta boundary), confirming that the MCMV was located on the surface of the coverslip. (b) The MCMV is sectioned completely in five, 70 nm-thick serial sections, having a height of about 350 nm on the coverslip. See Supplemental Movie 4 for higher magnification view of aligned images and the numerous vesicles and tubules inside.

**
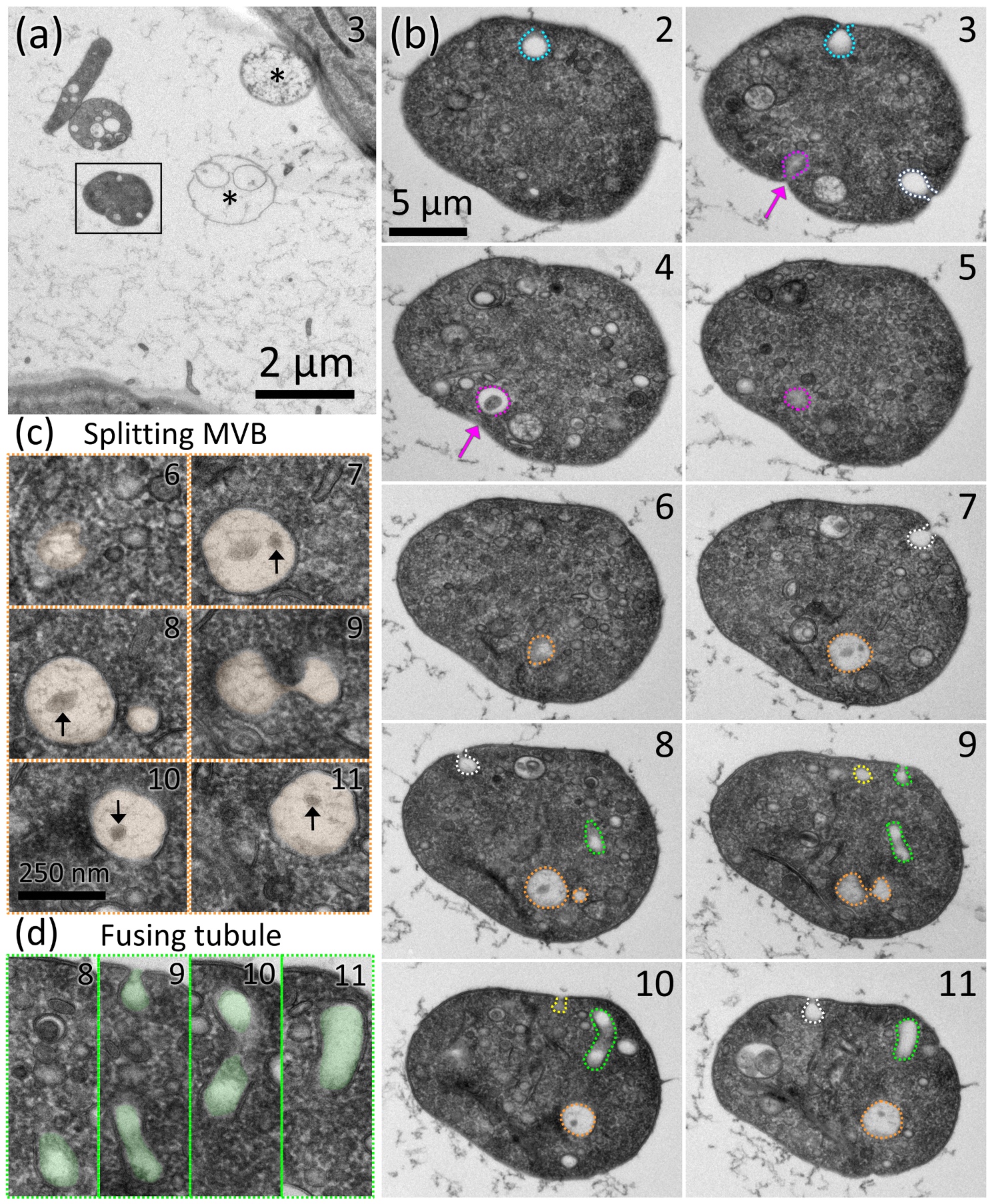
**

**SUPPLEMENTAL FIGURE 6:** (a) Field of view showing the location on the coverslip of the MCMV in Figure 3a (black box). Asterisks mark two structures that appear to be degraded MCMVs. Serial section number is indicated in the upper right corner of each image. (b) Ten sections through the MCMV boxed in (a). White dashed lines indicate omega figures that appear on one section. Turquoise and yellow dashed lines trace empty omega figures that span two sections. The magenta dashed lines trace an ILV-containing omega figure that spans three sections (magenta arrow indicates pore opening). Orange dashed lines outline an MVB-like organelle that contains four ILVs and appears to be splitting into two MVB-like organelles and is shown enlarged to the left in (c). Green dashed lines trace a curved tubule that goes in and out of sections 8-11, and in section 9 can be seen fusing with the MCMV periphery and is shown enlarged to the left in (d). Scalebar in (c) also applies to (d). Supplemental Movie 6 shows 11 aligned sections through the MCMV shown in (b).


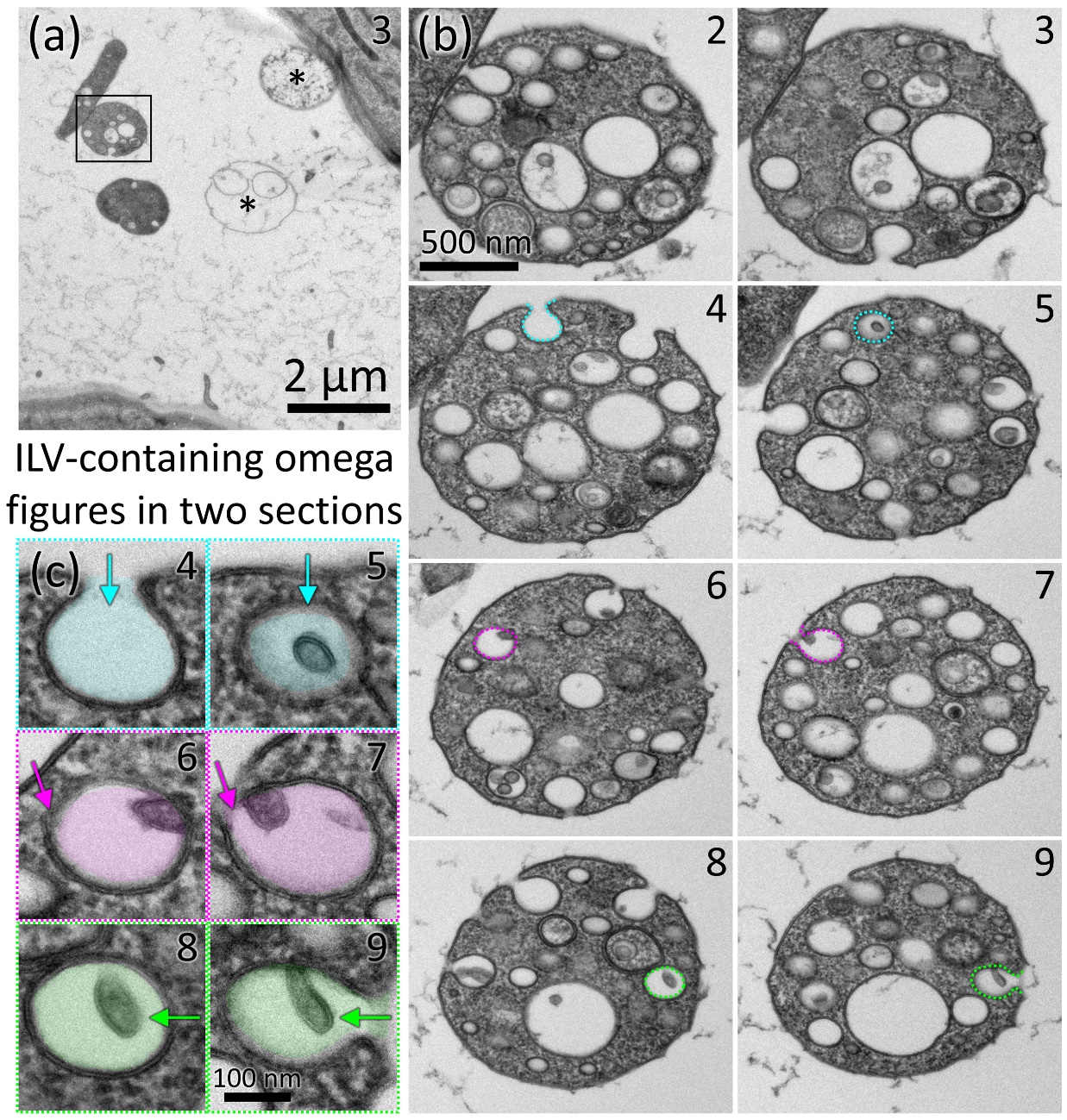


**SUPPLEMENTAL FIGURE 7:** (a) Field of view of the MCMV shown in Figure 3d-f (black box). The MCMV is near a tubule-shaped organelle that looks like an MCMV (not analyzed). Asterisks mark two structures that appear to be degraded MCMVs. Serial section number is indicated in the upper right corner. (b) Eight serial sections thought the MCMV. Colored dashed lines trace omega fusion figures that span two consecutive sections demonstrating that an omega figure that appears empty in once section can contain an ILV (or second ILV) in the neighboring section. (c) Enlarged views of the color-coded omega figures shown in (b). Arrows indicate the position of the opening to the omega figure. Supplemental Movie 7 shows 11 aligned sections through the MCMV shown in (b).

**
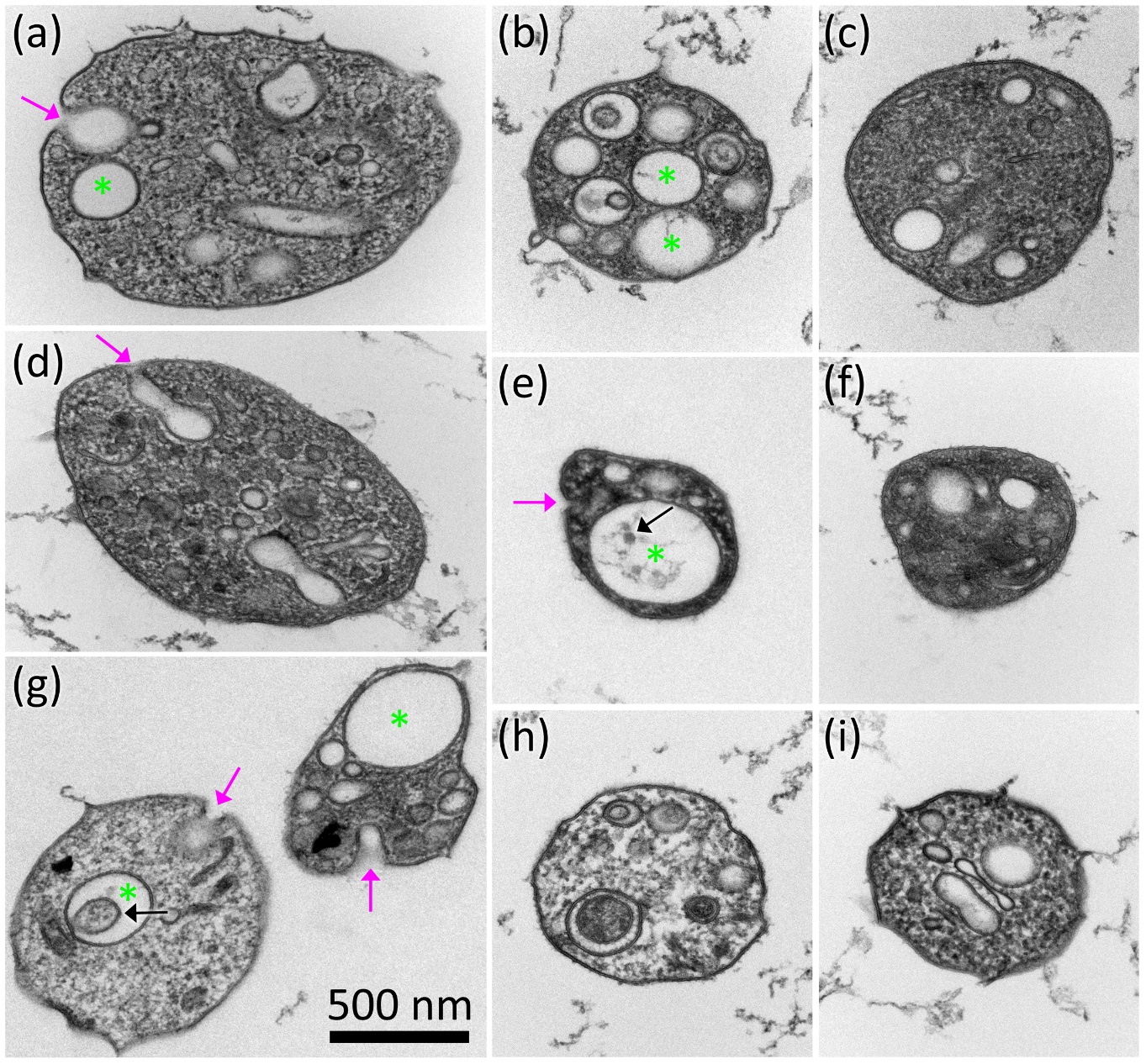
**

**SUPPLEMENTAL FIGURE 8:** Gallery of MCMVs preserved by freeze substitution. (a-i) Single thin sections of individual MCMVs are shown. (a, b, e, g) MCMVs containing MVB-like vesicles that have electron lucent lumens, and diameters over 200 nm (green asterisks), two of which contain ILV-like vesicles (black arrows in (e) and (g)). (a, d, e, g) MCMVs with omega figures on their limiting membranes (pink arrows).

**SUPPLEMENTAL MOVIES**

**SUPPLEMENTAL MOVIE 1:** Aligned stack of serial sections of the protrusion site shown in Figure 1. Sixteen serial sections encompassing 1120 nm thickness in Z-height beginning at the surface of the coverslip. Note that section 9 is absent due to a fold in that section. Section number is indicated in the upper right corner of each image.

**SUPPLEMENTAL MOVIE 2:** Aligned stack of serial sections of the protrusion site shown in Supplemental Figure 2. Seventeen sections encompassing 1190 nm thickness in Z-height. Section number is indicated in the upper right corner of each image.

**SUPPLEMENTAL MOVIE 3:** Aligned stack of serial sections of the protrusion site shown in Supplemental Figure 3. Odd-numbered sections encompassing 1050 nm thickness in Z-height are shown. Section number is indicated in the upper right corner of each image.

**SUPPLEMENTAL MOVIE 4:** Aligned stack of five serial sections capturing the entire height of the MCMV shown in Supplemental Figure 5 containing round and tubular internal vesicles. Section number is indicated in the upper right corner of each image.

**SUPPLEMENTAL MOVIE 5:** Aligned stack of ten serial sections capturing the entire height of the MCMV shown in Figure 2. Section number is indicated in the upper right corner of each image.

**SUPPLEMENTAL MOVIE 6:** Aligned stack of eleven serial sections through the MCMV shown in Figure 3a and Supplemental Figure 6b. Many internal vesicles are smaller than the 70 nm thickness of the section, and thus appear in only one section. Section number is indicated in the upper right corner of each image.

**SUPPLEMENTAL MOVIE 7:** Aligned stack of eleven serial sections through the MCMV shown in Figure 3d-f, and Supplemental Figure 7b, showing numerous omega figures occurring on the MCMV periphery. Section number is indicated in the upper right corner of each image.
